# Orthostatic blood pressure reactions and resting heart rate in relation to lung function - the Swedish CArdioPulmonary bioImage Study (SCAPIS)

**DOI:** 10.1186/s12890-024-03398-8

**Published:** 2024-11-28

**Authors:** Andreas Casselbrant, Christian Zambach, Artur Fedorowski, Gunnar Engström, Per Wollmer, Viktor Hamrefors

**Affiliations:** 1 Department of Clinical Sciences, Lund University, Malmö, Sweden; 2 Department of Ophthalmology, Skåne University Hospital, Lund, Sweden; 3 Department of Internal Medicine, Skåne University Hospital, Lund, Sweden; 4 Department of Cardiology, Karolinska University Hospital, Stockholm, Sweden; 5 Department of Translational Medicine, Lund University, Malmö, Sweden; 6 Department of Medical Imaging and Physiology, Skåne University Hospital, Malmö, Sweden; 7 Department of Cardiology, Skåne University Hospital, Malmö, Sweden

**Keywords:** Autonomic dysfunction, Orthostatic hypotension, Resting heart rate, COPD, Pulmonary function, SCAPIS

## Abstract

**Background:**

There is a well-known comorbidity between chronic obstructive pulmonary disease (COPD) and coronary artery disease (CAD) which is only partially explained by common risk factors. Markers of cardiovascular autonomic dysfunction (CVAD), such as orthostatic hypotension and increased resting heart rate, are strongly associated with CAD. The autonomic nervous system also innervates the airways, and several studies have shown an association between autonomic dysfunction and COPD. However, less is known about whether CVAD and impairment of respiratory capacity are related in the population. We thus aimed to assess the relationship between markers of subtle CVAD and lung function in middle-aged subjects.

**Methods:**

In this cross-sectional study, we analysed data from CVAD assessment (orthostatic blood pressure and heart rate measurements) and pulmonary function tests from 5886 individuals from the Swedish CArdioPulmonary bioImage Study (SCAPIS). Subjects were middle aged and randomly selected from the Swedish population. Linear regression models and ANOVA analyses were used to relate orthostatic blood pressure and resting heart rate to lung function parameters (forced vital capacity (FVC), forced expiratory volume in one second (FEV_1_), FEV_1_/FVC-ratio, diffusion capacity for carbon monoxide (D_LCO_), respiratory resistance at 5 Hz (R5), respiratory resistance at 20 Hz (R20), decrease in resistance from R5 to R20 (R5-R20), reactance in distal airways (X5), resonant frequency (Fres) and reactance area (AX)).

**Results:**

Increasing systolic orthostatic blood pressure, decreasing diastolic orthostatic blood pressure, and increased resting heart rate associated with lower FVC (all *p* < 0.001) and FEV_1_ (*p* = 0.001; *p* = 0.005; *p* < 0.001, respectively) in models including age, sex and height. Apart from diastolic orthostatic blood pressure and FEV_1_, all relationships remained significant after adjustment for possible confounders. Increased resting heart rate was associated with reduced D_LCO_ (*p* < 0.001).

**Conclusions:**

Increasing systolic orthostatic blood pressure, decreasing diastolic orthostatic blood pressure, and increased resting heart rate are associated with lower lung function, after adjustments for age, sex and height. These finding indicates associations between signs of cardiovascular autonomic dysfunction and lower lung function in the general population. However, the observed differences in lung function were small and the clinical application is unclear.

**Supplementary Information:**

The online version contains supplementary material available at 10.1186/s12890-024-03398-8.

## Introduction

The well known comorbidity between chronic obstructive pulmonary disease (COPD) and coronary artery disease (CAD) is not fully understood. Known common risk factors and risk markers, e.g. smoking, systemic inflammation, air pollution and genetics, do not fully explain this relationship. The autonomic nervous system (ANS) is highly involved within the cardiovascular and respiratory systems [1]. Among other functions, the ANS is responsible for homeostasis of blood pressure, heart rate and regulation of airway smooth muscle tone. Dysfunction of the cardiovascular aspects of the autonomic nervous system, cardiovascular autonomic dysfunction (CVAD), may result in orthostatic hypotension [2] and increased resting heart rate [3].

CVAD is a common feature of and a risk marker for CAD [4]. The ANS also plays an important role in homeostasis of the respiratory tract. It innervates smooth muscle, vasculature and glands in the airways [1], thereby regulating respiration as well as inflammation and microbial defence [5, 6]. Thus, subtle signs of CVAD, such as impaired orthostatic responses and reduced heart rate variability, may be a marker even for respiratory (autonomic) dysfunction. Studies have indeed shown a relationship between CVAD and COPD [7, 8] as well as reduced lung function in a population with a high proportion of smokers [9]. However, less is known about these relationships in individuals from the general population.

We tested the hypothesis that markers of CVAD are associated with lower lung function in the population. The hypothesis was tested by assessing several lung function parameters in relation to orthostatic blood pressure reactions and resting heart rate in middle-aged individuals from the general population.

## Methods

### Study population

**Fig. 1:**
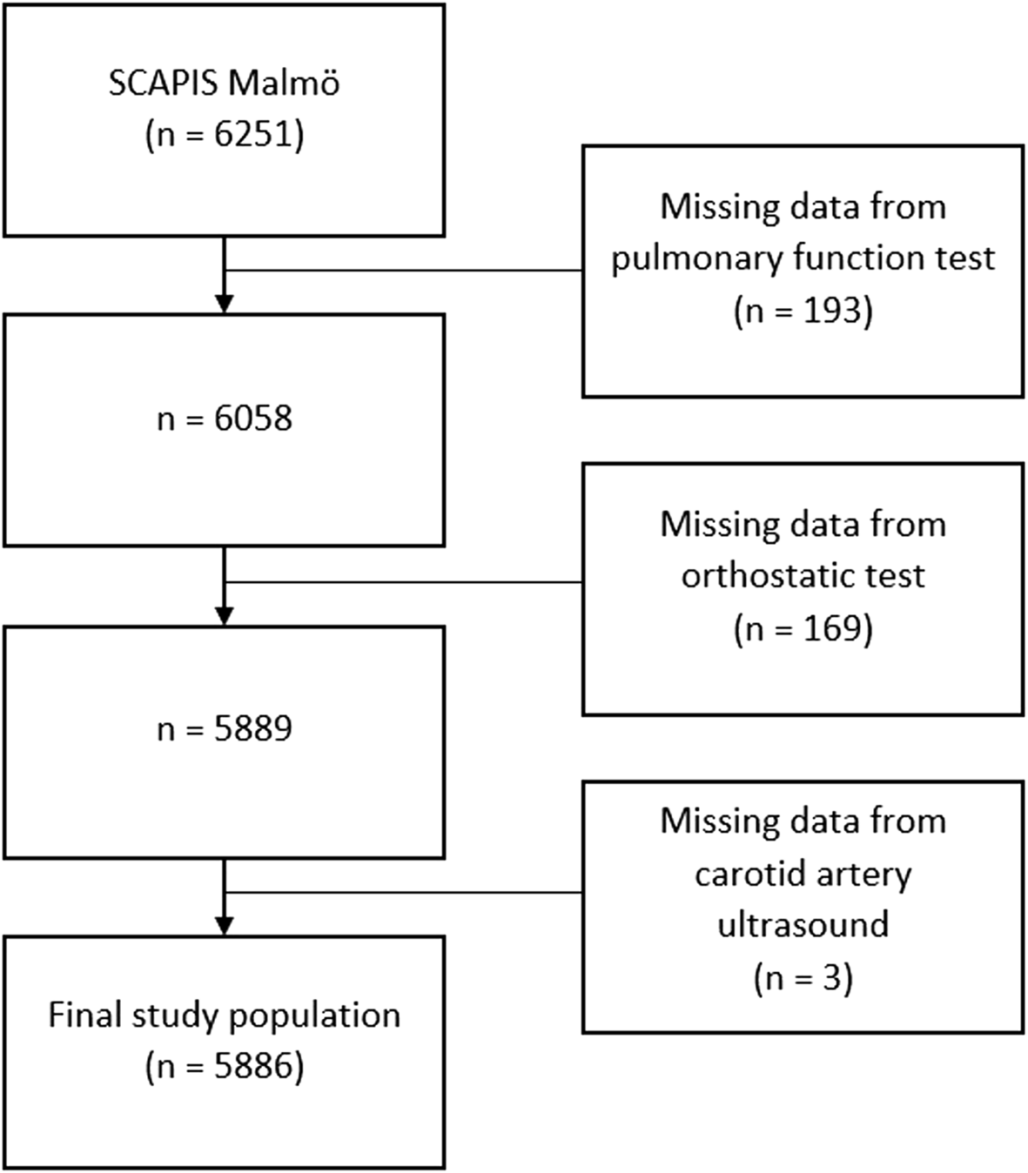
Selection of study participants for the current study. SCAPIS = Swedish CArdioPulmonary BioImage Study

The study data were derived from the Swedish CArdioPulmonary bioImage Study (SCAPIS). Detailed information regarding the SCAPIS cohort has been published [10]. Briefly, the SCAPIS study includes men and women aged 50–64, randomly selected from the Swedish population register. Examinations were carried out at six university hospitals in Sweden (Gothenburg, Malmö, Linköping, Stockholm, Umeå and Uppsala). Orthostatic blood pressure was included in the study protocol for participant at the Malmö screening centre and this subcohort was used for the present study. A total of 6251 subjects (participation rate 53%) took part in SCAPIS Malmö. Subjects without complete data from pulmonary function test, orthostatic test and ultrasound of the carotid arteries were excluded, leaving 5886 subjects in the final study population (Fig. 1).

### Patient characteristics

Information regarding smoking, antihypertensive drugs, use of β-blockers, diabetes and medication for COPD or asthma were acquired from a self-reported questionnaire. Questions regarding antihypertensive drugs and inhalation medication for COPD or asthma concerned use in the last two weeks. Data on participants use of β-blockers was retrieved from responds to the question *write down the names of your medications*. As for smoking status, subjects were asked to choose between *Yes*,* I smoke regularly*, *Yes*,* I smoke sometimes*, *No*,* I stopped smoking [year]*, and *No*,* I have never smoked*. For this study, the first two alternatives were defined as *Current smoking* and the latter two as *Non-smoking*. Subjects answering that they stopped smoking less than one year ago were considered current smokers. Variables age and height were used as continuous variables. Subjects that were either currently smoking or with a history of smoking were considered ever smokers.

### Orthostatic test and heart rate measurement

Supine blood pressure was measured in both arms using an automatic device (Omron M6. Omron Health Care Co. Kyoto. Japan). The two measurements from the arm with the highest blood pressure were used to calculate mean systolic (SBP) and diastolic (DBP) blood pressure. Supine blood pressure was recorded twice after 5 min of rest, with a one-minute break between measurements. Orthostatic blood pressure was measured twice after 3 min of standing. Orthostatic SBP and DBP reactions were defined as supine blood pressure minus standing blood pressure, meaning that a positive value corresponded to a decrease in orthostatic blood pressure.

Resting heart rate was measured with an OMRON automatic blood pressure monitor in the arm with the highest systolic blood pressure, on the same visit as the orthostatic test was performed.

### Pulmonary function test

All subjects performed dynamic spirometry and impulse oscillometry (IOS), including forced expiratory volume in 1 sec (FEV_1_), forced vital capacity (FVC), diffusing capacity for carbon monoxide (D_LCO_), respiratory resistance at 5 Hz (R5), at 20 Hz (R20), the decrease in resistance from R5 to R20 (R5-R20), reactance in distal airways (X5), resonant frequency (Fres) and reactance area (AX). Subjects inhaled 400 µg of salbutamol 15 min before examination. All references to spirometry or IOS in this study refer to measurements made post bronchodilation. Jaeger MasterScreen PFT (Carefusion, Hoechberg, Germany) was used in all measurements. Spirometry was performed with the subject sitting up and wearing a nose clip. Except for gas analyser linearity checks for D_LCO_ measurements, guidelines from European Respiratory Society (ERS) and American Thoracic Society (ATS) were followed in all spirometry examinations [11, 12].

Impulse oscillometry was used in all subjects in order to more specifically characterize a possible lung function impairment linked to CVAD. IOS was previously shown to associate with increased respiratory burden in individuals with normal spirometry in our cohort [13]. This method assesses pulmonary resistance (R) and reactance (X), which are the two components of respiratory impedance. It is measured by applying sound waves superimposed on the subject’s normal breathing. The impedance is then calculated as the ratio between pressure and flow due to the sound waves, separated from the tidal breathing. Resistance to sound waves of 5 Hz (R5) frequency is often considered to represent resistance in central and peripheral airways, and waves of 20 Hz frequency to represent resistance in central airways. Consequently, the difference between R5 and R20 (R5-R20) is considered an indicator of peripheral airway dysfunction. Reactance (X) reflects the elastance and inertance of the respiratory system. At 5 Hz, elastance dominates and X5 is often considered to reflect the properties of distal air spaces. Fres is the sound wave frequency at which the reactance is 0. When sound wave frequency is plotted against impedance, AX is the area above the curve between X5 and Fres. Except for performing one examination per subject instead of three, guidelines from Oostveen et al. [14] were followed. A previous study [15] has shown minimal difference between performing one and three IOS examinations.

### Coronary calcium score

Coronary artery calcium images were received using a dual-source CT scanner with a Stellar Detector (Somatom Definition Flash, Siemens Medical Solutions), details have been published elsewhere [16]. Coronary calcium levels were assessed according to Agatston score [17], and coronary artery calcium score was calculated according to international standards [18]. Patients were administered metoprolol for control of heart rate.

### Carotid artery plaque

Presence of carotid plaque was assessed using a Siemens Acuson S2000 ultrasound scanner equipped with a 9L4 linear transducer (both from Siemens, Forchheim, Germany). Presence of plaques was defined as “a focal structure encroaching into the arterial lumen of at least 0.5 mm or 50% of the surrounding IMT (intima-media thickness) value, or demonstrates a thickness > 1.5 mm as measured from the intima-lumen interface to the media-adventitia interface”, according to Mannheim Carotid Intima-Media Thickness and Plaque Consensus [19].

### Statistical analyses

Linear regression models were used to assess the relationship between cardiovascular autonomic function measurements (SBP reaction, DBP reaction, and resting heart rate) and lung function measurements (FVC, FEV_1_, FEV_1_/FVC, D_LCO_, R5, R20, R5-20, X5, Fres and Ax). Autonomic function measurements were used as independent variables and lung function measurements as dependent variables in separate models. The linear regression models included three different adjustment levels; Basic model (*age*, *sex*, *height*); Adjustment model 1 (Basic model plus *current smoking*); Adjustment model 2 (Basic model plus *current smoking*, *supine SBP*, *carotid artery plaques*, *coronary calcium score*, *antihypertensive drugs*, *use of β-blockers*, *diabetes*,* inhalation medication for either COPD or asthma*). Analyses using adjustment model 2 included 5522 subjects after removal of participants with no data on calcium score (*n* = 364).

To allow adjustment for age, sex, and length of both lung function parameters and markers of CVAD in linear regression analyses, absolute values ​​of FVC and FEV_1_ were used. However, lung function parameters in Table 1 are reported in absolute values and in percent of predicted.

**Table 1:** Population characteristics

	All patients (*n*=5886)
Age, years	57.5 (4.3)
Sex, female	53.1
BMI, kg·m^−2^	27.2 (4.6)
Smoking status	
Current smoker	17.4
Ex-smoker	39.5
Never-smoker	43.1
Orthostatic test	
Supine SBP, mmHg	122.7 (16.5)
Orthostatic SBP, mmHg decrease	-3.8 (10.3)
Supine DBP, mmHg	76.5 (9.7)
Orthostatic DBP, mmHg decrease	-9.5 (6.4)
Resting heart rate, min^-1^	60.9 (9.0)
Spirometry	
FVC, ml	4065 (981)
FVC, % predicted	100.6 (14.2)
FEV_1_, ml	3184 (772)
FEV_1_, % predicted	100.5 (13.0)
FEV_1_/FVC ratio	0.770 (0.069)
FEV_1_/FVC < 0,7	8.8
D_LCO_, mmol/(min*kPa)	8.0 (1.8)
D_LCO_, % predicted	98.9 (15.1)
Impulse oscillometry	
R5, kPa/(L/s)	0.31 (0.12)
R5, % predicted	108.8 (33.6)
R20, kPa/(L/s)	0.28 (0.10)
R20, % predicted	123.9 (37.7)
R5 – R20, kPa/(L/s)	0.03 (0.05)
R5 – R20, % predicted	N/A
X5, kPa/(L/s)	-0.08 (0.05)
X5, % predicted	89.5 (42.8)
Fres, Hz	9.86 (4.27)
Fres, % predicted	81.9 (28.6)
AX, Kpa/L	0.18 (0.22)
AX, % predicted	64.8 (67.1)
Medical history	
Diabetes	4.9
Using hypertensive medication	20.5
Using β-blockers	18.5
Inhalation medication for COPD/asthma	4.8
Carotid artery plaques, yes	60.7
Calcium score, ≥ 1	42.2

Presented as mean (SD) or % of total. IOS parameters presented as median (interquartile range)

Percent predicted values for spirometry parameters ​​were obtained using the Global Lung Function Initiative (GLI) equation. The GLI equation takes ethnicity into account when calculating percent predictive values. We do not have data regarding the ethnicity of study participants. Therefore, all our study participants were considered Caucasians, since this is the ethnicity of a large majority of the SCAPIS cohort.

Reference equations by Schulz et al. [20] were used to calculate the percent predicted values for IOS parameters. Because of skewed distribution and some variables with both negative and positive measurements, medians and interquartile ranges are reported instead of means for absolute and percent predicted IOS parameters in Table 1.

In linear regression models, orthostatic SBP reaction, orthostatic DBP reaction and resting heart rate were analysed as continuous variables. Dichotomous variables were created for *carotid artery plaques (yes; no)* and *coronary calcium score (0; ≥ 1)*.

In addition to linear regression analyses, subjects were divided into quartiles based on their SBP reaction, DBP reaction and resting heart rate, separately (creating quartiles ortSBP1-4, ortDBP1-4 and HR1-4). Quartiles 4, i.e. ortSBP4 and ortDBP4 included participants with the lowest ability to maintain blood pressure during an orthostatic test (i.e. greatest decrease in orthostatic SBP and DBP, respectively), whereas HR4 included subjects with the highest resting heart rate. ANOVA models were used to analyse difference in lung function parameters between quartiles. The ANOVA test involved parametric testing and the *p*-values denote overall ANOVA *p*-value for differences between the groups.

Chi-squared test were performed in order to compare quartiles ortSBP1-4, ortDBP1-4 and HR1-4 according to chronic airway obstruction (FEV_1_/FVC < 0.7), current smoking or ever smoking.

In order to account for a potential interaction of sex on the independent variables, we performed multiplicative interaction analyses between sex and SBP reaction, DBP reaction and resting heart rate on FVC, FEV_1_ and D_LCO_ respectively.

Normal distribution was assessed by visual inspection of histograms and the use of parametric tests were further justified by the high number of included participants.

All analyses were carried out using SPSS Statistics version 25 (IBM Corp., Armonk, NY, USA).

## Results

Study population characteristics are listed in Table 1.

Results from chi-squared test on differences in chronic airway obstruction and smoking status between quartiles ortSBP1-4, ortDBP1-4 and HR1-4 are listed in Supplementary Table 1. Chronic airway obstruction (FEV_1_/FVC < 0.7) was more common in HR4 than in other HR-quartiles (*p* = 0.021). A history of smoking was more common in ortSBP1 than in other ortSBP-quartiles (*p* = 0.022). Apart from these associations, quartiles did not significantly differ regarding chronic airway obstruction, current smoking or ever smoking.

### SBP reaction in relation to lung function

**Fig. 2:**
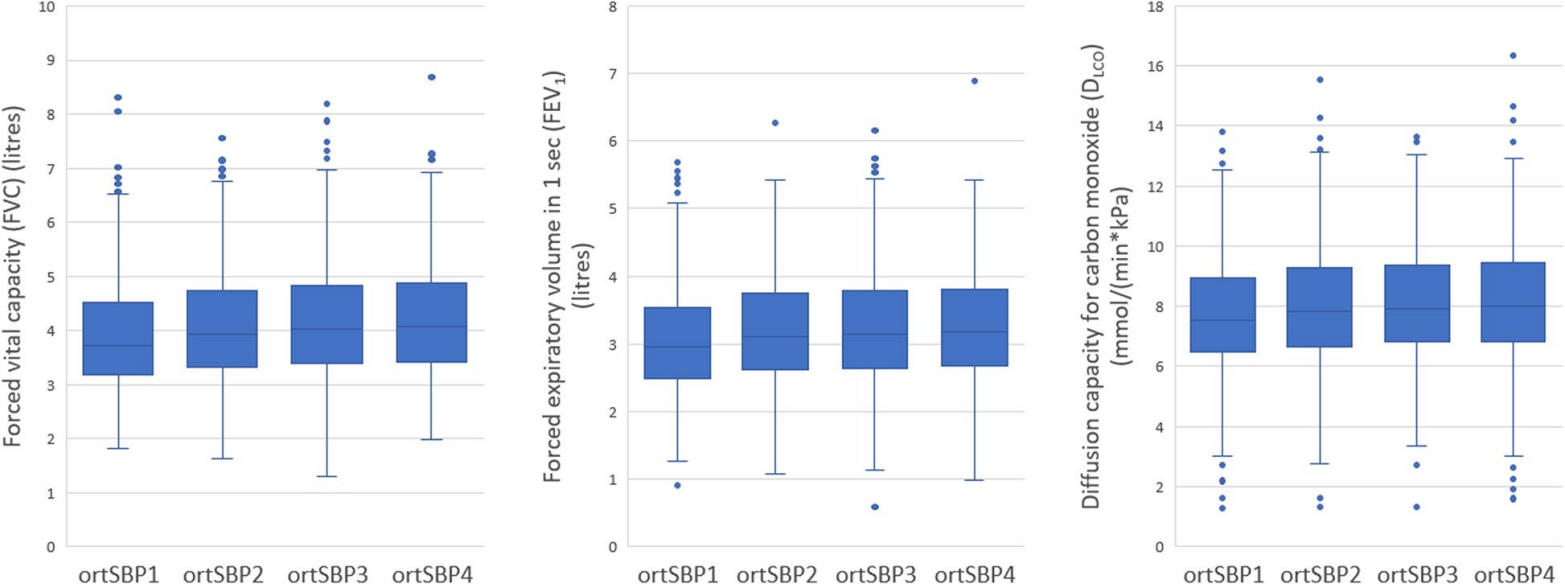
Box plots of FVC, FEV_1_ and D_LCO_ according to quartiles of orthostatic systolic blood pressure reaction. *P*-values denote overall ANOVA (parametric test) differences. FVC by ortSBP group (unadjusted ANOVA: *p* < 0.001). FEV_1_ by ortSBP group (unadjusted ANOVA *p* < 0.001). D_LCO_ by ortSBP group (unadjusted ANOVA *p* < 0.001)

Cut-offs and mean systolic blood pressure reactions for quartiles ortSBP1-4 are listed in Table 2. Linear regression models (Table 3) and ANOVA analyses (Fig. 2) revealed lower FVC and FEV_1_ in subjects with increasing orthostatic SBP. In linear regression models (adjusted for age, sex and height), SBP increase of 10mmHg when standing up corresponded with 26 ml and 20 ml lower FVC and FEV_1_ respectively, compared to those with no change in SBP during orthostatic test. In unadjusted ANOVA-analyses, D_LCO_ was lower in ortSBP groups with increasing orthostatic SBP (*P* < 0.001), however not in adjusted linear regression analyses. There were no associations between FEV_1_/FVC-ratio and SBP reaction.

**Table 2:** Quartiles based on SBP reaction during orthostatic test

	n	Cut-off SBP reaction	Mean SBP decrease
ortSBP1	1471	-66.0 ­– -10.5	-16.5
ortSBP2	1505	-10.0 – -4.0	-6.8
ortSBP3	1452	-3.5 – 2.5	-0.7
ortSBP4	1425	3.0 – 47.5	9.2

Blood pressure reaction was defined as the difference between supine blood pressure and orthostatic blood pressure (supine blood pressure subtracted by orthostatic blood pressure). Thus, a negative blood pressurereaction represents an increased blood pressure during the orthostatic test

**Table 3:** Linear regression analysis - orthostatic SBP reaction in relation to spirometry lung function parameters

	Basic model			Adjustment model 1			Adjustment model 2		
	β	R^2^	*p*-value	β	R^2^	*p*-value	β	R^2^	*p*-value
FVC	2.64	0.71	**<0.001**	2.53	0.71	**<0.001**	2.92	0.70	**0.004**
FEV_1_	1.95	0.65	**0.001**	1.59	0.66	**0.007**	2.55	0.65	**0.003**
D_LCO_	0.003	0.55	0.090	0.002	0.58	0.211	0.000	0.57	0.875

A positive β represents a positive correlation between the lung function parameter and orthostatic SBP reaction (e.g increasing FVC with decreasing orthostatic SBP)

β = The increase in FVC/FEV_1_/D_LCO_ for every 1 mmHg reduction in orthostatic SBP

FVC (ml); FEV_1_ (ml); D_LCO_ (mmol/(min*kPa))

Basic model (adjusted for age, sex and height)

Adjustment model 1 (age, sex, height, current smoking)

Adjustment model 2 (age, sex, height, current smoking, supine SBP, carotid artery plaques, coronary calcium score, antihypertensive drugs, β-blockers, diabetes, inhalation medication for COPD or asthma)

For IOS, subjects with increasing orthostatic SBP had increased pulmonary resistance and decreased pulmonary reactance (linear regression: R5, *p* < 0.001; R20, *p* = 0.002; R5-R20, *p* = 0.112; X5, *p* < 0.001; Fres, *p* < 0.001; AX, *p* < 0.001. All using Adjustment model 2) (Supplementary Table 2).

### DBP reaction in relation to lung function

**Fig. 3:**
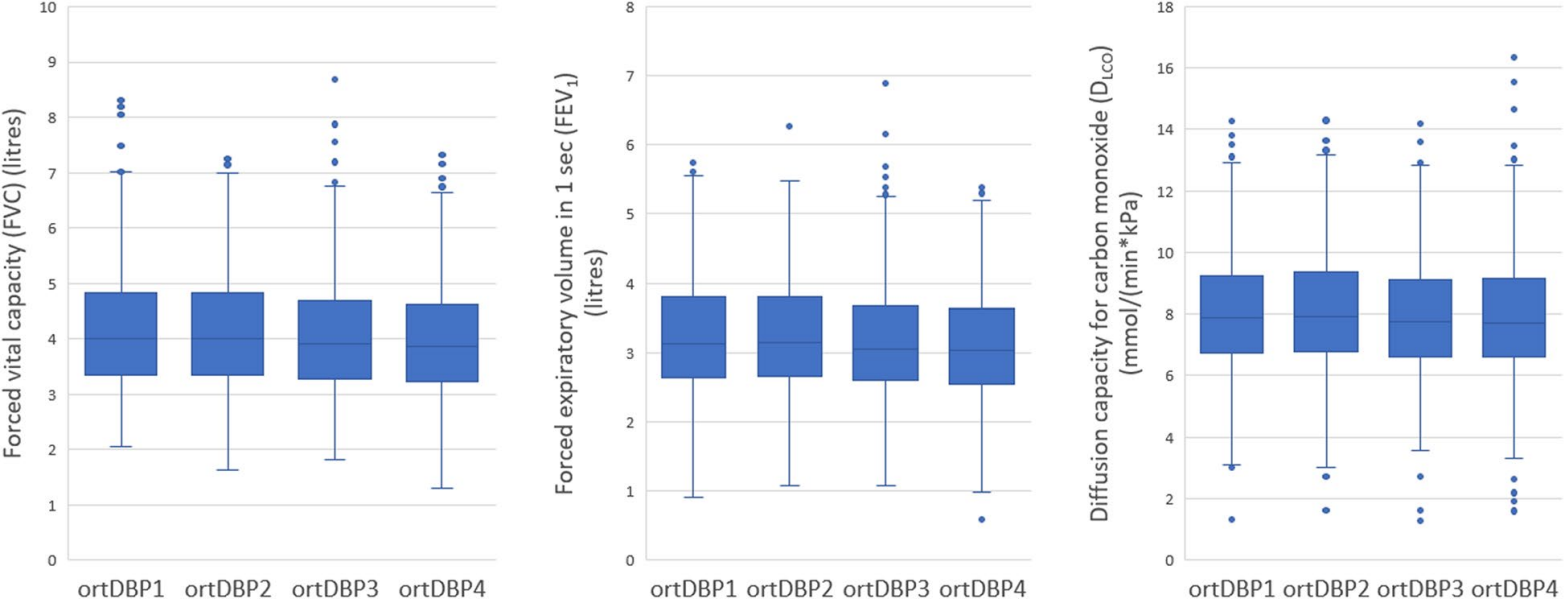
Box plots of FVC, FEV_1_ and D_LCO_ according to quartiles of orthostatic diastolic blood pressure reaction. *P*-values denote overall ANOVA (parametric test) differences. FVC by ortDBP group (unadjusted ANOVA *p* < 0.001). FEV_1_ by ortSBP group (unadjusted ANOVA *p* < 0.001). D_LCO_ by ortDBP group (unadjusted ANOVA *p* = 0.011)

Cut-offs and mean diastolic blood pressure reactions for each quartile are listed in Table 4. Subjects with a minor increase, and with stable or decreasing DBP during orthostatic test (i.e. DBP3 and DBP4) had lower lung function parameters, compared to subjects with a more prominent increase in orthostatic DBP (Table 5; Fig. 3). In linear regression models (adjusted for age, sex and height), DBP decrease of 10mmHg when standing up corresponded with 43ml and 26ml lower FVC and FEV_1_ respectively, compared to those with no change in DBP during orthostatic test. There were no associations between FEV_1_/FVC-ratio and orthostatic DBP reaction in linear regression models or ANOVA-analyses.

**Table 4:** Quartiles based on DBP reaction during orthostatic test

	n	Cut-off DBP reaction	Mean DBP decrease
ortDBP1	1470	-38.5 – -14.0	-17.4
ortDBP2	1621	-13.5 – -9.5	-11.4
ortDBP3	1367	-9.0 – -5.5	-7.3
ortDBP4	1394	-5.0 – 26.5	-1.1

Blood pressure reaction was defined as the difference between orthostatic blood pressure and supine blood pressure (Supine blood pressure subtracted by orthostatic blood pressure). Thus, a negative blood pressure reaction represents an increased blood pressure during the orthostatic test

**Table 5:** Linear regression analysis - orthostatic DBP reaction in relation to spirometry lung function parameters

	Basic model				Adjustment model 1			Adjustment model 2		
	β		R^2^	*p*-value	β	R^2^	*p*-value	β	R^2^	*p*-value
FVC	-4.26		0.71	**<0.001**	-4.26	0.71	**<0.001**	-3.92	0.70	**0.014**
FEV_1_	-2.59		0.65	**0.005**	-2.62	0.66	**0.005**	-2.28	0.65	0.097
D_LCO_	-0.002		0.55	0.496	-0.001	0.58	0.719	-0.006	0.57	0.526

A negative β represents a negative correlation between the lung function parameter and orthostatic DBP reaction (e.g. decreasing FVC with decreasing orthostatic DBP)

β = The increase in FVC/FEV_1_/D_LCO_ for every 1 mmHg decrease in orthostatic DBP reaction

FVC (ml); FEV_1_ (ml); D_LCO_ (mmol/(min*kPa))

Basic model (adjusted for age, sex and height)

Adjustment model 1 (age, sex, height, current smoking)

Adjustment model 2 (age, sex, height, current smoking, supine SBP, carotid artery plaques, coronary calcium score, antihypertensive drugs, β-blockers, diabetes, inhalation medication for COPD or asthma)

Decreasing orthostatic DBP was associated with increased X5 and decreased Fres (Linear regression: X5, *p* = 0.015; Fres, *p* = 0.002) (Supplementary Table 3). However, there was no significant difference in mean R5, R20, R5-R20, X5, Fres or AX between groups ortDBP1-4 (ANOVA: R5, *p* = 0.322; R20, *p* = 0.481; R5-R20, *p* = 0.631; X5, *p* = 0.964; Fres, *p* = 0.063; AX, *p* = 0.548).

### Resting heart rate in relation to lung function

**Fig. 4:**
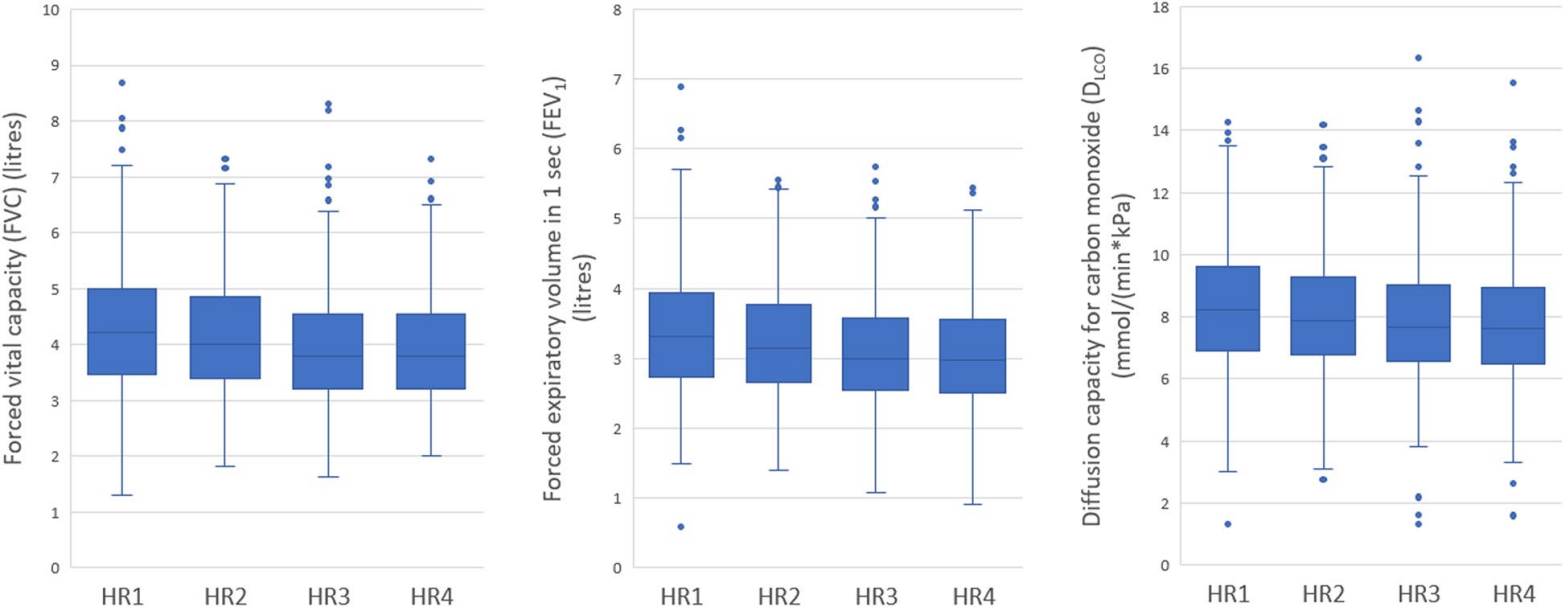
Box plots of FVC, FEV_1_ and D_LCO_ according to quartiles of resting heart rate. *P*-values denote overall ANOVA (parametric test) differences. FVC by HR group (unadjusted ANOVA *p* < 0.001). FEV_1_ by HR group (unadjusted ANOVA *p* < 0.001). D_LCO_ by HR group (unadjusted ANOVA *p* < 0.001)

Cut-offs and mean resting heart rate for each quartile are listed in Table 6. Increased resting heart rate was associated with decreased FVC, FEV_1_, D_LCO_ and FEV_1_/FVC-ratio (Table 7; Fig. 4).

**Table 6:** Quartiles based on resting heart rate

	n	Cut-off resting heart rate	Mean resting heart rate
HR1	1422	37 – 54	50.1
HR2	1614	55 – 60	57.6
HR3	1268	61 – 65	62.9
HR4	1580	66 - 108	72.3

**Table 7:** Linear regression analysis – resting heart rate in relation to spirometry lung function parameters

	Basic model			Adjustment model 1			Adjustment model 2		
	β	R^2^	*p*-value	β	R^2^	*p*-value	β	R^2^	*p*-value
FVC	-6.39	0.71	**<0.001**	-6.45	0.71	**<0.001**	-6.07	0.70	**<0.001**
FEV_1_	-6.16	0.66	**<0.001**	-6.12	0.66	**<0.001**	-6.04	0.66	**<0.001**
D_LCO_	-0.010	0.55	**<0.001**	-0.010	0.58	**<0.001**	-0.015	0.58	**<0.001**

β = The increase in FVC/FEV_1_/D_LCO_ for every 1 bpm increase in resting heart rate

FVC (ml); FEV_1_ (ml); D_LCO_ (mmol/(min*kPa))

Basic model (adjusted for age, sex and height)

Adjustment model 1 (age, sex, height, current smoking)

Adjustment model 2 (age, sex, height, current smoking, supine SBP, carotid artery plaques, coronary calcium score, antihypertensive drugs, β-blockers, diabetes, inhalation medication for COPD or asthma)

Linear regression revealed a significant association between increased heart rate and reduced FEV_1_/FVC-ratio (β − 0.27% points per 10 beats/min increase in heart rate, *p* = 0.006). This association was also significant in Adjustment model 1 (*p* = 0.014) and Adjustment model 2 (*p* = 0.003). ANOVA-analyses showed no significant difference in mean FEV_1_/FVC-ratio between groups HR1 – HR4.

Subjects with elevated resting heart rate had higher resistance and lower reactance on IOS testing, compared to subjects with lower resting heart rate (Linear regression: R5, *p* < 0.001; R20, *p* = 0.011; R5-R20, *p* < 0.001; X5, *p* < 0.001 ; Fres, *p* < 0.001 ; AX, *p* < 0.001. All using Adjustment model 2) (Supplementary Table 4).

### Sub-group analyses

The results from subgroup analyses of current smokers and non-smokers were similar to those from the entire study population. More information regarding subgroup analyses is provided in Supplementary Tables 5–7 (non-smokers) and 8–10 (smokers).

Separate analyses in subjects with and without inhalation medication are shown in Supplementary Tables 11-13 and 14–16.

There were significant interactions (*p* < 0.001) for sex and HR on FEV_1_ and FVC. Accordingly, sex-stratified analyses are presented in Supplementary Tables 17 and 18. There were no other interactions for sex and the independent variables on FEV_1_, FVC or DLCO.

### Additional analyses

Unadjusted ANOVA analyses were also performed on lung function measurements expressed as percent predicted. These resulted in significant differences in mean percent predicted FVC (*p* < 0.001), FEV_1_ (*p* < 0.001), D_LCO_ (*p* < 0.001) between ortSBP groups, FVC (*p* < 0.001), FEV_1_ (*p* < 0.001), D_LCO_ (*p* = 0.011) between ortDBP groups, and FVC (*p* < 0.001), FEV_1_ (*p* < 0.001), D_LCO_ (*p* < 0.001) between HR groups.

## Discussion

We observed that an increase in systolic blood pressure and a decrease in diastolic blood pressure during orthostatic provocation, as well as higher resting heart rate, were associated with lower FVC and FEV_1_ in the general population. These relationships were independent of cardiovascular risk factors, including smoking, prevalence of coronary atherosclerosis and diabetes.

A pronounced drop in blood pressure upon standing is a major sign of autonomic failure and even subtle impaired orthostatic responses may indicate subclinical CVAD [2]. In our study, decreased orthostatic DBP was associated with worse lung function, which is in line with earlier studies [9]. Standing up leads to pooling of blood below the diaphragm and thereby a reduction in venous return and cardiac output. This activates baroreceptors in the aortic arch and carotid arteries, which increases sympathetic stimulation to the heart and vessels, restoring blood pressure [2]. The association between decreasing orthostatic DBP and worse lung function seen in our study may in part be caused by the impaired baroreceptor sensitivity, which has been previously noticed in patients with COPD [21].

Heart rate is regulated by the parasympathetic nervous system [22] and autonomic dysfunction is associated with increased heart rate [3]. There is evidence [21] for sympathetic over activation in individuals with COPD, possibly caused by hypoxia, increased inflammation and increased intrathoracic pressure. A higher sympathetic activity may be one explanation to the increased heart rate seen in patients with worse lung function in our study, even if subclinical.

In opposite to DBP, increasing SBP during orthostatic provocation was strongly associated with worse lung function. These results are not in line with previous results from our group [9], in which orthostatic decrease in SBP associated with worse lung function. Data in that study were derived from Malmö Preventive Project (MPP). Differences in study design and subjects’ characteristics may explain the contradictory results. Compared to SCAPIS, MPP had a substantially higher proportion of smokers and overall a worse cardiovascular risk profile, including more orthostatic hypotension. Also worth noting, PFT in MPP were performed without bronchodilation.

There are a number of possible hypotheses that may explain why increasing SBP during standing is associated with worse lung function. Increasing orthostatic SBP may be caused by excess neurohormonal activation [23], including norepinephrine [24]. The increased levels of plasma norepinephrine may in turn be caused by the sympathetic over activation in individuals with subclinically reduced lung function.

Increased orthostatic blood pressure has also been associated with increased arterial stiffness [23]. Arterial stiffness can cause increased SBP and decreased DBP (increased pulse pressure) [25] and is related to COPD [26, 27]. However, in our analyses we adjusted for several risk factors for arterial stiffness [28], such as age, diabetes, hypertensive medication, atherosclerosis (calcium score and carotid artery plaques), but could despite this observe a strong association between rising SBP on standing and worse lung function. Another argument against arterial stiffness as explanation can be seen when comparing quartiles one and four. There was no increase in pulse pressure or marked decrease in diastolic blood pressure in ortSBP1, as would be expected if subjects in this group had increased prevalence of arterial stiffness.

The relationships between SBP reaction and IOS parameters followed the same tendencies as the relationships between SBP reaction and FVC, FEV_1_ and D_LCO_; subjects with increasing SBP during an orthostatic test had worse lung function than those with stable or decreasing SBP. An elevated resting heart rate was also associated with worse IOS lung function parameters.

The relationships between DBP reaction and IOS parameters are more uncertain. There was no significant relationship between DBP reaction and R5, R20 and R5-R20. Subjects with decreasing DBP during orthostatic test had improved X5, Fres and AX, compared to subjects with increasing or stable DBP. However, when comparing means of X5, Fres and AX between quartiles ortDBP1-4, unadjusted ANOVA-analyses revealed no significant associations.

The overall aim of this study was to explore CVAD as a possible link in the early stages of the pathophysiology for COPD and CAD. Although, a causal relationship between COPD, CAD and CVAD is impossible to demonstrate using cross-sectional design, there is evidence for sympathetic over activation in individuals with COPD, which in turn may lead to endothelial dysfunction, arterial stiffness and cardiovascular autonomic dysfunction [21]. Thus, it is possible that CVAD (and subsequently CAD) is a consequence of COPD rather than a risk factor for it.

Only 1.8% (*n* = 104) of subjects in our study had manifest orthostatic hypotension, defined as orthostatic SBP decrease of ≥ 20 mmHg and/or DBP decrease of ≥ 10 mmHg. As indicated by our previous findings [9] results may be different in a population with a worse cardiovascular risk profile, including higher prevalence of orthostatic hypotension. A pronounced increase and decrease in systolic blood pressure during orthostatic provocation represent the extremes of a pathological reaction, and recent studies in SCAPIS have shown that higher orthostatic systolic blood pressure seems to associate with subclinical atherosclerosis to a similar magnitude as excessive orthostatic BP decrease [29]. It may be that increased systolic orthostatic BP represents an earlier phenotype of cardiovascular aging compared to decreasing systolic orthostatic BP, possibly explaining the association with worse lung function in our generally healthy population.

We observed a greater decrease in FVC and FEV_1_ with increasing resting heart rate in men compared to women, as shown by significant interactions. We have not found any previous studies addressing specifically the interaction between sex and resting heart rate on lung function. Studies investigating this interaction on mortality from cardiovascular disease and all-cause mortality have had conflicting results [30].

Smoking is a risk factor for both COPD [31] and cardiovascular autonomic dysfunction [32]. In our study, subgroup analyses of smokers and non-smokers showed essentially the same results as analyses on all subjects. In line with this, a review by Mohammed et al. conclude that cigarette smoking does not influence autonomic function in COPD [7].

The function of the autonomic nervous system can be improved by interventions such as physical activity [33]. Whether or not CVAD is a causal factor for compromised lung function, that may be targeted for interventions, remains to be further investigated in future studies.

### Limitations

Our study has a number of limitations.

Invitation to SCAPIS was conducted through informational brochure and telephone calls, and the inclusion rate was 53%. This entails a risk of selection bias since healthier individuals tend to accept invitation to a greater extent, a phenomenon known as the “healthy cohort effect”. Still, the risk factors and characteristics of the SCAPIS participants and the target population can be considered reasonably similar [34].

Resting heart rate is used as a marker of cardiovascular autonomic dysfunction in our study. However, other factors such as physical status [35] also effect resting heart rate. Symptoms and comorbidities from pulmonary disease lead to physical deconditioning [36]. According to Bahrainy et al., the change in resting heart rate from physical activity is likely a result of intrinsic electrophysiological changes in the sinus node and not due to changes in the autonomic nervous system [35]. Thus, the association between lung function and resting heart rate described in our study may be caused more so by physical inactivity rather than CVAD.

It should be noted that one in five in the current study population had beta blockers whereas only one in twenty used inhalations. Such medications may affect both orthostatic blood pressure, heart rate and lung function measurements, however, adjusting for these variables did not significantly change the associations. We do not have any data regarding participants use of antihypertensive medication on the same day as their orthostatic blood pressure measurement.

Finally, the observed differences in lung function were small in absolute terms and may not translate into clinically meaningful differences in this overall healthy cohort. We believe that larger differences should be sought in patient materials with a higher prevalence of impaired lung function. Naturally, the association between CVAD at baseline and incident COPD would have been of interest. However, the follow-up time in SCAPIS is only a few years on average and future studies may address this association.

## Conclusion

Increased systolic blood pressure and decreased diastolic blood pressure during orthostatic provocation, as well as increased resting heart rate, are associated with lower lung function in the general population, after accounting for age, sex, height and cardiovascular risk factors. These results suggest that signs of cardiovascular autonomic dysfunction may be associated with lower lung function in subjects without overt cardiac or respiratory disease, but their clinical application is currently unclear.

## Supplementary Information

Supplementary Material 1.

## Data Availability

The datasets used and/or analysed during the current study are available after application to the SCAPIS steering committee (www.scapis.org).
