# Supplementary material for: Orthostatic blood pressure reactions and resting heart rate in relation to lung function - the Swedish CArdioPulmonary bioImage Study (SCAPIS)
Source: BMC Pulm Med. 2024 Nov 28;24:587. doi: 10.1186/s12890-024-03398-8 (PMC11603930; doi:10.1186/s12890-024-03398-8)
Supplement: Supplementary file 1 — Supplementary Material 1. [file 12890_2024_3398_MOESM1_ESM.docx]

**Supplementary Results**

| Supplementary Table 1 | | | |
| --- | --- | --- | --- |
| Chi^2^-test – Differences between ortSBP groups 1-4, ortDBP groups 1-4 and HR groups 1-4 regarding airway obstruction (FEV1/FVC <0.7) and smoking | | | |
|  | FEV1/FVC <0.7 | Current smoking | Ever smoking |
|  | p-value | p-value | p-value |
| ortSBP group | 0.257 | 0.079 | **0.022** |
| ortDBP group | 0.094 | 0.984 | 0.142 |
| HR group | **0.021** | 0.065 | 0.475 |

**SBP reaction in relation to IOS parameters**
In linear regression models, subjects with increasing orthostatic SBP had increased R5, R20, R5-R20, Fres and AX and decreased X5, i.e. worse lung function, compared to subjects with stable or decreasing SBP (Supplementary table 2).
Means of IOS lung function parameters (R5, R20, R5-R20, X5, Fres and AX) were all significantly different between groups ortSBP1 - ortSBP4 (ANOVA-analyses, p<0.001 for all). The same tendencies were seen with IOS parameters as with spirometry parameters, with ortSBP1-2 having worse lung function than ortSBP3-4 (higher mean R5, R20, R5-R20, Fres and AX, and lower X5).

| Supplementary Table 2 | | | | | | | | | |
| --- | --- | --- | --- | --- | --- | --- | --- | --- | --- |
| Linear regression analyses – orthostatic SBP reaction in relation to impulse oscillometry lung function parameters | | | | | | | | | |
|  | Basic model | | | Adjustment model 1 | | | Adjustment model 2 | | |
|  | β | R^2^ | p-value | β | R^2^ | p-value | β | R^2^ | p-value |
| R5 | -0.0005 | 0.19 | **<0.001** | -0.0004 | 0.20 | **<0.001** | -0.0006 | 0.22 | **<0.001** |
| R20 | -0.0003 | 0.21 | **0.005** | -0.0003 | 0.22 | **0.006** | -0.0004 | 0.22 | **0.002** |
| R5-R20 | -0.0002 | 0.04 | **<0.001** | -0.0002 | 0.05 | **0.003** | -0.0002 | 0.08 | 0.112 |
| X5 | 0.0003 | 0.19 | **<0.001** | 0.0003 | 0.19 | **<0.001** | 0.0004 | 0.23 | **<0.001** |
| Fres | -0.0245 | 0.09 | **<0.001** | -0.0222 | 0.09 | **<0.001** | -0.0262 | 0.14 | **<0.001** |
| AX | -0.0020 | 0.09 | **<0.001** | -0.0018 | 0.10 | **<0.001** | -0.0021 | 0.15 | **<0.001** |

A positive β represents a positive correlation between the lung function parameter and orthostatic SBP reaction
β = The increase in the lung function parameter for every 1 mmHg reduction in orthostatic SBP
R5 (kPa/(L/s)); R20 (kPa/(L/s)); R5-R20 (kPa/(L/s)); X5 (kPa/(L/s)); Fres (Hz); AX (kPa/L)
β adjusted for age, sex, height
Basic model (adjusted for age, sex and height)
Adjustment model 1 (age, sex, height, current smoking)
Adjustment model 2 (age, sex, height, current smoking, supine SBP, carotid artery plaques, coronary calcium score, antihypertensive drugs, β-blockers, diabetes, inhalation medication for COPD or asthma)

**DBP reaction in relation to IOS parameters**

Linear regression analyses on the relationship between DBP reaction and parameters from impulse oscillometry are listed in Supplementary Table 3.

There were no significant differences in ANOVA-analyses comparing means of IOS lung function parameters (R5, R20, R5-R20, X5, Fres and AX) between groups ortDBP1-4 (data not shown).

| Supplementary Table 3 | | | | | | | | | |
| --- | --- | --- | --- | --- | --- | --- | --- | --- | --- |
| Linear regression analyses – orthostatic DBP reaction in relation to impulse oscillometry lung function parameters | | | | | | | | | |
|  | Basic model | | | Adjustment model 1 | | | Adjustment model 2 | | |
|  | β | R^2^ | p-value | β | R^2^ | p-value | β | R^2^ | p-value |
| R5 | 0.0001 | 0.19 | 0.510 | 0.0001 | 0.20 | 0.643 | -0.0003 | 0.21 | 0.215 |
| R20 | 0.0001 | 0.21 | 0.454 | 0.0001 | 0.22 | 0.632 | -0.0002 | 0.22 | 0.328 |
| R5-R20 | 0.0000 | 0.04 | 0.890 | 0.0000 | 0.05 | 0.861 | -0.0001 | 0.08 | 0.411 |
| X5 | 0.0002 | 0.19 | **0.024** | 0.0002 | 0.19 | **0.033** | 0.0004 | 0.23 | **0.015** |
| Fres | -0.0204 | 0.09 | **0.003** | -0.0200 | 0.09 | **0.004** | -0.0319 | 0.13 | **0.002** |
| AX | -0.0014 | 0.09 | **0.042** | -0.0014 | 0.09 | **0.043** | -0.0020 | 0.15 | 0.051 |

A positive β represents a positive correlation between the lung function parameter and orthostatic DBP reaction
β = The increase in the lung function parameter for every 1 mmHg reduction in orthostatic DBP
R5 (kPa/(L/s)); R20 (kPa/(L/s)); R5-R20 (kPa/(L/s)); X5 (kPa/(L/s)); Fres (Hz); AX (kPa/L)
β adjusted for age, sex, height
Basic model (adjusted for age, sex and height)
Adjustment model 1 (age, sex, height, current smoking)
Adjustment model 2 (age, sex, height, current smoking, supine SBP, carotid artery plaques, coronary calcium score, antihypertensive drugs, β-blockers, diabetes, inhalation medication for COPD or asthma)

**Resting heart rate in relation to IOS parameters**

Linear regression analyses on the relationships between resting heart rate and parameters from impulse oscillometry are listed in Supplementary Table 4. Elevated resting heart rate was associated with increased R5, R20, R5-R20, Fres, Ax and decreased X5.

ANOVA-analyses comparing mean R5, R20, R5-R20, X5, Fres and AX in groups HR1-4 all showed a significant difference between groups (p < 0.001 for all). Subjects with increased resting heart rate, HR3-4, had worse lung function than HR1-2 (higher mean R5, R20, R5-R20, Fres, AX and lower mean X5).

| Supplementary Table 4 | | | | | | | | | |
| --- | --- | --- | --- | --- | --- | --- | --- | --- | --- |
| Linear regression analyses – resting heart rate in relation to impulse oscillometry lung function parameters | | | | | | | | | |
|  | Basic model | | | Adjustment model 1 | | | Adjustment model 2 | | |
|  | β | R^2^ | p-value | β | R^2^ | p-value | β | R^2^ | p-value |
| R5 | 0.0009 | 0.20 | **<0.001** | 0.0009 | 0.20 | **<0.001** | 0.0007 | 0.22 | **<0.001** |
| R20 | 0.0005 | 0.22 | **<0.001** | 0.0005 | 0.22 | **<0.001** | 0.0004 | 0.22 | **0.011** |
| R5-R20 | 0.0004 | 0.05 | **<0.001** | 0.0004 | 0.05 | **<0.001** | 0.0002 | 0.08 | **0.028** |
| X5 | -0.0004 | 0.19 | **<0.001** | -0.0004 | 0.19 | **<0.001** | -0.0004 | 0.23 | **<0.001** |
| Fres | 0.0317 | 0.09 | **<0.001** | 0.0318 | 0.10 | **<0.001** | 0.0261 | 0.14 | **<0.001** |
| AX | 0.0030 | 0.09 | **<0.001** | 0.0031 | 0.10 | **<0.001** | 0.0025 | 0.15 | **<0.001** |

A positive β represents a positive correlation between the lung function parameter and resting heart rate
β = The increase in the lung function parameter for every 1 bpm increase in heart rate
R5 (kPa/(L/s)); R20 (kPa/(L/s)); R5-R20 (kPa/(L/s)); X5 (kPa/(L/s)); Fres (Hz); AX (kPa/L)
β adjusted for age, sex, height
Basic model (adjusted for age, sex and height)
Adjustment model 1 (age, sex, height, current smoking)
Adjustment model 2 (age, sex, height, current smoking, supine SBP, carotid artery plaques, coronary calcium score, antihypertensive drugs, β-blockers, diabetes, inhalation medication for COPD or asthma)

**Sub-group analyses: Smokers and non-smokers**

Linear regression analyses on the relationship between markers of CVAD and lung function parameters are presented in Table 5-7 (non-smokers, n = 4750) and 8-10 (smokers, n = 999).

| Supplementary Table 5 | | | | | | |
| --- | --- | --- | --- | --- | --- | --- |
| Linear regression analyses – SBP reaction in relation to lung function parameters in non-smokers (n = 4750) | | | | | | |
|  | Basic model | | | Adjustment model 2 | | |
|  | β | R^2^ | p-value | β | R^2^ | p-value |
| FVC | 2.44 | 0.71 | **0.001** | 2.66 | 0.70 | **0.017** |
| FEV_1_ | 1.53 | 0.67 | **0.016** | 2.24 | 0.66 | **0.017** |
| D_LCO_ | 0.0013 | 0.58 | 0.446 | -0.0009 | 0.58 | 0.716 |
| R5 | -0.0004 | 0.20 | **<0.001** | -0.0006 | 0.22 | **0.004** |
| R20 | -0.0002 | 0.23 | **0.038** | -0.0004 | 0.24 | **0.011** |
| R5-R20 | -0.0002 | 0.04 | **0.001** | -0.0002 | 0.07 | 0.100 |
| X5 | 0.0003 | 0.19 | **<0.001** | 0.0004 | 0.25 | **<0.001** |
| Fres | -0.0240 | 0.08 | **<0.001** | -0.0268 | 0.12 | **<0.001** |
| AX | -0.0019 | 0.09 | **<0.001** | -0.0024 | 0.15 | **<0.001** |

A positive β represents a positive correlation between the lung function parameter and orthostatic SBP reaction
β = The increase in the lung function parameter for every 1 mmHg reduction in orthostatic SBP
FVC (ml); FEV_1_ (ml); D_LCO_ (mmol/(min*kPa)); R5 (kPa/(L/s)); R20 (kPa/(L/s)); R5-R20 (kPa/(L/s)); X5 (kPa/(L/s)); Fres (Hz); AX (kPa/L)
Basic model (adjusted for age, sex and height)
Adjustment model 2 (age, sex, height, supine SBP, carotid artery plaques, coronary calcium score, antihypertensive drugs, β-blockers, diabetes, inhalation medication for COPD or asthma)

| Supplementary Table 6 | | | | | | |
| --- | --- | --- | --- | --- | --- | --- |
| Linear regression analyses – DBP reaction in relation to lung function parameters in non-smokers (n = 4750) | | | | | | |
|  | Basic model | | | Adjustment model 2 | | |
|  | β | R^2^ | p-value | β | R^2^ | p-value |
| FVC | -3.47 | 0.71 | **0.004** | -4.03 | 0.70 | **0.021** |
| FEV_1_ | -1.87 | 0.67 | 0.065 | -2.17 | 0.66 | 0.140 |
| D_LCO_ | 0.0006 | 0.58 | 0.835 | -0.0048 | 0.58 | 0.220 |
| R5 | 0.0001 | 0.20 | 0.586 | -0.0003 | 0.22 | 0.380 |
| R20 | 0.0001 | 0.22 | 0.456 | -0.0001 | 0.23 | 0.658 |
| R5-R20 | 0.0000 | 0.04 | 0.938 | -0.0002 | 0.07 | 0.350 |
| X5 | 0.0003 | 0.19 | **0.013** | 0.0005 | 0.24 | **0.002** |
| Fres | -0.0251 | 0.08 | **<0.001** | -0.0360 | 0.12 | **0.001** |
| AX | -0.0019 | 0.09 | **0.009** | -0.0029 | 0.15 | **0.004** |

A positive β represents a positive correlation between the lung function parameter and orthostatic DBP reaction
β = The increase in the lung function parameter for every 1 mmHg reduction in orthostatic DBP
FVC (ml); FEV_1_ (ml); D_LCO_ (mmol/(min*kPa)); R5 (kPa/(L/s)); R20 (kPa/(L/s)); R5-R20 (kPa/(L/s)); X5 (kPa/(L/s)); Fres (Hz); AX (kPa/L)
Basic model (adjusted for age, sex and height)
Adjustment model 2 (age, sex, height, supine SBP, carotid artery plaques, coronary calcium score, antihypertensive drugs, β-blockers, diabetes, inhalation medication for COPD or asthma)

| Supplementary Table 7 | | | | | | |
| --- | --- | --- | --- | --- | --- | --- |
| Linear regression analyses – resting heart rate in relation to lung function parameters in non-smokers (n = 4750) | | | | | | |
|  | Basic model | | | Adjustment model 2 | | |
|  | β | R^2^ | p-value | β | R^2^ | p-value |
| FVC | -6.69 | 0.71 | **<0.001** | -5.47 | 0.70 | **<0.001** |
| FEV_1_ | -5.77 | 0.67 | **<0.001** | -5.15 | 0.67 | **<0.001** |
| D_LCO_ | -0.0079 | 0.59 | **<0.001** | -0.0106 | 0.59 | **<0.001** |
| R5 | 0.0009 | 0.20 | **<0.001** | 0.0007 | 0.22 | **<0.001** |
| R20 | 0.0005 | 0.23 | **<0.001** | 0.0005 | 0.24 | **0.002** |
| R5-R20 | 0.0003 | 0.04 | **<0.001** | 0.0002 | 0.07 | 0.117 |
| X5 | -0.0004 | 0.19 | **<0.001** | -0.0004 | 0.24 | **<0.001** |
| Fres | 0.0273 | 0.08 | **<0.001** | 0.0240 | 0.12 | **0.003** |
| AX | 0.0028 | 0.09 | **<0.001** | 0.0024 | 0.15 | **0.001** |

A positive β represents a positive correlation between the lung function parameter and resting heart rate
β = The increase in the lung function parameter for every 1 bpm increase in heart rate
FVC (ml); FEV_1_ (ml); D_LCO_ (mmol/(min*kPa)); R5 (kPa/(L/s)); R20 (kPa/(L/s)); R5-R20 (kPa/(L/s)); X5 (kPa/(L/s)); Fres (Hz); AX (kPa/L)
Basic model (adjusted for age, sex and height)
Adjustment model 2 (age, sex, height, supine SBP, carotid artery plaques, coronary calcium score, antihypertensive drugs, β-blockers, diabetes, inhalation medication for COPD or asthma)

| Supplementary Table 8 | | | | | | |
| --- | --- | --- | --- | --- | --- | --- |
| Linear regression analyses – SBP reaction in relation to lung function parameters in smokers (n = 999) | | | | | | |
|  | Basic model | | | Adjustment model 2 | | |
|  | β | R^2^ | p-value | β | R^2^ | p-value |
| FVC | 2.58 | 0.69 | 0.129 | 0.0026 | 0.69 | 0.313 |
| FEV_1_ | 1.25 | 0.60 | 0.409 | 0.0018 | 0.60 | 0.440 |
| D_LCO_ | 0.0041 | 0.48 | 0.327 | -0.0024 | 0.46 | 0.724 |
| R5 | -0.0005 | 0.18 | 0.103 | -0.0006 | 0.18 | 0.198 |
| R20 | -0.0004 | 0.17 | **0.048** | -0.0005 | 0.16 | 0.165 |
| R5-R20 | 0.0000 | 0.06 | 0.920 | -0.0001 | 0.10 | 0.732 |
| X5 | 0.0003 | 0.18 | 0.050 | 0.0002 | 0.19 | 0.586 |
| Fres | -0.0124 | 0.10 | 0.256 | -0.0153 | 0.15 | 0.424 |
| AX | -0.0007 | 0.10 | 0.583 | 0.0002 | 0.15 | 0.927 |

A positive β represents a positive correlation between the lung function parameter and orthostatic SBP reaction
β = The increase in the lung function parameter for every 1 mmHg reduction in orthostatic SBP
FVC (ml); FEV_1_ (ml); D_LCO_ (mmol/(min*kPa)); R5 (kPa/(L/s)); R20 (kPa/(L/s)); R5-R20 (kPa/(L/s)); X5 (kPa/(L/s)); Fres (Hz); AX (kPa/L)
Basic model (adjusted for age, sex and height)
Adjustment model 2 (age, sex, height, supine SBP, carotid artery plaques, coronary calcium score, antihypertensive drugs, β-blockers, diabetes, inhalation medication for COPD or asthma)

| Supplementary Table 9 | | | | | | |
| --- | --- | --- | --- | --- | --- | --- |
| Linear regression analyses – DBP reaction in relation to lung function parameters in smokers (n = 999) | | | | | | |
|  | Basic model | | | Adjustment model 2 | | |
|  | β | R^2^ | p-value | β | R^2^ | p-value |
| FVC | -8.50 | 0.69 | **0.002** | -5.30 | 0.69 | 0.182 |
| FEV_1_ | -6.71 | 0.61 | **0.006** | -5.04 | 0.60 | 0.171 |
| D_LCO_ | -0.0086 | 0.48 | 0.202 | -0.0186 | 0.46 | 0.080 |
| R5 | 0.0000 | 0.18 | 0.960 | -0.0006 | 0.18 | 0.414 |
| R20 | -0.0001 | 0.16 | 0.709 | -0.0006 | 0.15 | 0.322 |
| R5-R20 | 0.0001 | 0.06 | 0.628 | 0.0000 | 0.10 | 0.955 |
| X5 | -0.0001 | 0.18 | 0.775 | -0.0003 | 0.19 | 0.469 |
| Fres | 0.0051 | 0.10 | 0.776 | -0.0017 | 0.15 | 0.957 |
| AX | 0.0010 | 0.10 | 0.622 | 0.0034 | 0.15 | 0.360 |

A positive β represents a positive correlation between the lung function parameter and orthostatic DBP reaction
β = The increase in the lung function parameter for every 1 mmHg reduction in orthostatic DBP
FVC (ml); FEV_1_ (ml); D_LCO_ (mmol/(min*kPa)); R5 (kPa/(L/s)); R20 (kPa/(L/s)); R5-R20 (kPa/(L/s)); X5 (kPa/(L/s)); Fres (Hz); AX (kPa/L)
Basic model (adjusted for age, sex and height)
Adjustment model 2 (age, sex, height, supine SBP, carotid artery plaques, coronary calcium score, antihypertensive drugs, β-blockers, diabetes, inhalation medication for COPD or asthma)

| Supplementary Table 10 | | | | | | |
| --- | --- | --- | --- | --- | --- | --- |
| Linear regression analyses – resting heart rate in relation to lung function parameters in smokers (n = 999) | | | | | | |
|  | Basic model | | | Adjustment model 2 | | |
|  | β | R^2^ | p-value | β | R^2^ | p-value |
| FVC | -5.24 | 0.69 | **0.007** | -8.93 | 0.70 | **0.002** |
| FEV_1_ | -7.55 | 0.61 | **<0.001** | -9.90 | 0.61 | **<0.001** |
| D_LCO_ | -0.0185 | 0.49 | **<0.001** | -0.0367 | 0.49 | **<0.001** |
| R5 | 0.0008 | 0.18 | **0.013** | 0.0002 | 0.18 | 0.728 |
| R20 | 0.0002 | 0.17 | 0.342 | -0.0004 | 0.15 | 0.390 |
| R5-R20 | 0.0006 | 0.07 | **<0.001** | 0.0005 | 0.11 | 0.061 |
| X5 | -0.0005 | 0.18 | **0.002** | -0.0004 | 0.20 | 0.242 |
| Fres | 0.0533 | 0.12 | **<0.001** | 0.0347 | 0.15 | 0.105 |
| AX | 0.0044 | 0.11 | **0.001** | 0.0029 | 0.15 | 0.264 |

A positive β represents a positive correlation between the lung function parameter and resting heart rate
β = The increase in the lung function parameter for every 1 bpm increase in heart rate
FVC (ml); FEV_1_ (ml); D_LCO_ (mmol/(min*kPa)); R5 (kPa/(L/s)); R20 (kPa/(L/s)); R5-R20 (kPa/(L/s)); X5 (kPa/(L/s)); Fres (Hz); AX (kPa/L)
Basic model (adjusted for age, sex and height)
Adjustment model 2 (age, sex, height, supine SBP, carotid artery plaques, coronary calcium score, antihypertensive drugs, β-blockers, diabetes, inhalation medication for COPD or asthma)

**Sub-group analyses: Inhalation medication for asthma or COPD**

| Supplementary Table 11 | | | | | | | | | |
| --- | --- | --- | --- | --- | --- | --- | --- | --- | --- |
| Linear regression analysis - orthostatic SBP reaction in relation to spirometry lung function parameters – subjects without inhalation medication for asthma or COPD  (n = 5602) | | | | | | | | | |
|  | Basic model | | | Adjustment model 1 | | | Adjustment model 2 | | |
|  | Β | R^2^ | p-value | β | R^2^ | p-value | β | R^2^ | p-value |
| FVC | 2.78 | 0.70 | **<0.001** | 2.68 | 0.70 | **<0.001** | 2.83 | 0.69 | **0.008** |
| FEV_1_ | 1.90 | 0.66 | **0.001** | 1.56 | 0.67 | **0.008** | 2.17 | 0.66 | **0.015** |
| D_LCO_ | 0.003 | 0.55 | 0.120 | 0.002 | 0.58 | 0.263 | -0.001 | 0.58 | 0.686 |

A positive β represents a positive correlation between the lung function parameter and orthostatic SBP reaction
β = The increase in the lung function parameter for every 1 mmHg reduction in orthostatic SBP
FVC (ml); FEV_1_ (ml); D_LCO_ (mmol/(min*kPa))
Basic model (adjusted for age, sex and height)
Adjustment model 1 (age, sex, height, current smoking)
Adjustment model 2 (age, sex, height, current smoking, supine SBP, carotid artery plaques, coronary calcium score, antihypertensive drugs, β-blockers, diabetes)

| Supplementary Table 12 | | | | | | | | | |
| --- | --- | --- | --- | --- | --- | --- | --- | --- | --- |
| Linear regression analysis - orthostatic DBP reaction in relation to spirometry lung function parameters – subjects without inhalation medication for asthma or COPD  (n = 5602) | | | | | | | | | |
|  | Basic model | | | Adjustment model 1 | | | Adjustment model 2 | | |
|  | β | R^2^ | p-value | β | R^2^ | p-value | β | R^2^ | p-value |
| FVC | -4.05 | 0.70 | **<0.001** | -4.04 | 0.70 | **<0.001** | -3.67 | 0.69 | **0.031** |
| FEV_1_ | -2.51 | 0.66 | **0.007** | -2.53 | 0.67 | **0.007** | -2.29 | 0.66 | 0.107 |
| D_LCO_ | 0.000 | 0.55 | 0.855 | 0.000 | 0.58 | 0.848 | -0.004 | 0.58 | 0.276 |

A positive β represents a positive correlation between the lung function parameter and orthostatic DBP reaction
β = The increase in the lung function parameter for every 1 mmHg reduction in orthostatic DBP
FVC (ml); FEV_1_ (ml); D_LCO_ (mmol/(min*kPa))
Basic model (adjusted for age, sex and height)
Adjustment model 1 (age, sex, height, current smoking)
Adjustment model 2 (age, sex, height, current smoking, supine SBP, carotid artery plaques, coronary calcium score, antihypertensive drugs, β-blockers, diabetes)

| Supplementary Table 13 | | | | | | | | | |
| --- | --- | --- | --- | --- | --- | --- | --- | --- | --- |
| Linear regression analysis – resting heart rate in relation to spirometry lung function parameters – subjects without inhalation medication for asthma or COPD (n = 5602) | | | | | | | | | |
|  | Basic model | | | Adjustment model 1 | | | Adjustment model 2 | | |
|  | β | R^2^ | p-value | β | R^2^ | p-value | β | R^2^ | p-value |
| FVC | -5.97 | 0.71 | **<0.001** | -6.02 | 0.71 | **<0.001** | -5.49 | 0.70 | **<0.001** |
| FEV_1_ | -5.39 | 0.67 | **<0.001** | -5.37 | 0.67 | **<0.001** | -5.54 | 0.66 | **<0.001** |
| D_LCO_ | -0.009 | 0.56 | **<0.001** | -0.009 | 0.58 | **<0.001** | -0.014 | 0.58 | **<0.001** |

A positive β represents a positive correlation between the lung function parameter and resting heart rate
β = The increase in the lung function parameter for every 1 bpm increase in heart rate
FVC (ml); FEV_1_ (ml); D_LCO_ (mmol/(min*kPa))
Basic model (adjusted for age, sex and height)
Adjustment model 1 (age, sex, height, current smoking)
Adjustment model 2 (age, sex, height, current smoking, supine SBP, carotid artery plaques, coronary calcium score, antihypertensive drugs, β-blockers, diabetes)

| Supplementary Table 14 | | | | | | | | | |
| --- | --- | --- | --- | --- | --- | --- | --- | --- | --- |
| Linear regression analysis - orthostatic SBP reaction in relation to spirometry lung function parameters – subjects with inhalation medication for asthma or COPD  (n = 284) | | | | | | | | | |
|  | Basic model | | | Adjustment model 1 | | | Adjustment model 2 | | |
|  | β | R^2^ | p-value | β | R^2^ | p-value | β | R^2^ | p-value |
| FVC | 0.44 | 0.73 | 0.875 | 0.43 | 0.73 | 0.879 | 3.85 | 0.75 | 0.272 |
| FEV_1_ | 1.95 | 0.51 | 0.513 | 1.26 | 0.54 | 0.665 | 3.81 | 0.57 | 0.293 |
| D_LCO_ | 0.004 | 0.46 | 0.595 | 0.003 | 0.53 | 0.723 | 0.001 | 0.53 | 0.947 |

A positive β represents a positive correlation between the lung function parameter and orthostatic SBP reaction
β = The increase in the lung function parameter for every 1 mmHg reduction in orthostatic SBP
FVC (ml); FEV_1_ (ml); D_LCO_ (mmol/(min*kPa))
Basic model (adjusted for age, sex and height)
Adjustment model 1 (age, sex, height, current smoking)
Adjustment model 2 (age, sex, height, current smoking, supine SBP, carotid artery plaques, coronary calcium score, antihypertensive drugs, β-blockers, diabetes)

| Supplementary Table 15 | | | | | | | | | |
| --- | --- | --- | --- | --- | --- | --- | --- | --- | --- |
| Linear regression analysis - orthostatic DBP reaction in relation to spirometry lung function parameters – subjects with inhalation medication for asthma or COPD  (n = 284) | | | | | | | | | |
|  | Basic model | | | Adjustment model 1 | | | Adjustment model 2 | | |
|  | β | R^2^ | p-value | β | R^2^ | p-value | β | R^2^ | p-value |
| FVC | -8.60 | 0.73 | **0.045** | -8.59 | 0.74 | **0.046** | -7.00 | 0.75 | 0.153 |
| FEV_1_ | -5.96 | 0.51 | 0.195 | -6.21 | 0.54 | 0.165 | -4.70 | 0.57 | 0.352 |
| D_LCO_ | -0.026 | 0.47 | **0.036** | -0.027 | 0.54 | **0.017** | -0.031 | 0.55 | **0.018** |

A positive β represents a positive correlation between the lung function parameter and orthostatic DBP reaction
β = The increase in the lung function parameter for every 1 mmHg reduction in orthostatic DBP
FVC (ml); FEV_1_ (ml); D_LCO_ (mmol/(min*kPa))
Basic model (adjusted for age, sex and height)
Adjustment model 1 (age, sex, height, current smoking)
Adjustment model 2 (age, sex, height, current smoking, supine SBP, carotid artery plaques, coronary calcium score, antihypertensive drugs, β-blockers, diabetes)

| Supplementary Table 16 | | | | | | | | | |
| --- | --- | --- | --- | --- | --- | --- | --- | --- | --- |
| Linear regression analysis – resting heart rate in relation to spirometry lung function parameters – subjects with inhalation medication for asthma or COPD (n = 284) | | | | | | | | | |
|  | Basic model | | | Adjustment model 1 | | | Adjustment model 2 | | |
|  | β | R^2^ | p-value | β | R^2^ | p-value | β | R^2^ | p-value |
| FVC | -12.2 | 0.73 | **<0.001** | -12.0 | 0.73 | **<0.001** | -11.7 | 0.76 | **0.002** |
| FEV_1_ | -11.6 | 0.51 | **0.001** | -10.6 | 0.54 | **0.002** | -10.8 | 0.58 | **0.005** |
| D_LCO_ | -0.026 | 0.47 | **0.005** | -0.021 | 0.53 | **0.017** | -0.024 | 0.55 | **0.015** |

A positive β represents a positive correlation between the lung function parameter and resting heart rate
β = The increase in the lung function parameter for every 1 bpm increase in heart rate
FVC (ml); FEV_1_ (ml); D_LCO_ (mmol/(min*kPa))
Basic model (adjusted for age, sex and height)
Adjustment model 1 (age, sex, height, current smoking)
Adjustment model 2 (age, sex, height, current smoking, supine SBP, carotid artery plaques, coronary calcium score, antihypertensive drugs, β-blockers, diabetes)

**Sub-group analyses: Gender**

| Supplementary Table 17 | | | | | | | | | |
| --- | --- | --- | --- | --- | --- | --- | --- | --- | --- |
| Linear regression analysis – resting heart rate in relation to FVC and FEV_1_ in women (n = 3124) | | | | | | | | | |
|  | Basic model | | | Adjustment model 1 | | | Adjustment model 2 | | |
|  | β | R^2^ | p-value | Β | R^2^ | p-value | Β | R^2^ | p-value |
| FVC | -3.10 | 0.44 | **< 0.001** | -3.22 | 0.43 | **< 0.001** | -4.51 | 0.45 | **< 0.001** |
| FEV_1_ | -4.05 | 0.37 | **< 0.001** | -4.12 | 0.38 | **< 0.001** | -5.23 | 0.41 | **< 0.001** |

A positive β represents a positive correlation between the lung function parameter and resting heart rate
β = The increase in the lung function parameter for every 1 bpm increase in heart rate.
FVC (ml); FEV_1_ (ml)
Basic model (adjusted for age and height)
Adjustment model 1 (age, height, current smoking)
Adjustment model 2 (age, height, current smoking, supine SBP, carotid artery plaques, coronary calcium score, antihypertensive drugs, β-blockers, diabetes, inhalation medication for COPD or asthma)

| Supplementary Table 18 | | | | | | | | | |
| --- | --- | --- | --- | --- | --- | --- | --- | --- | --- |
| Linear regression analysis – resting heart rate in relation to FVC and FEV_1_ in men (n = 2762) | | | | | | | | | |
|  | Basic model | | | Adjustment model 1 | | | Adjustment model 2 | | |
|  | β | R^2^ | p-value | β | R^2^ | p-value | β | R^2^ | p-value |
| FVC | -9.61 | 0.43 | **< 0.001** | -9.61 | 0.43 | **< 0.001** | -7.57 | 0.45 | **< 0.001** |
| FEV_1_ | -8.22 | 0.36 | **< 0.001** | -8.04 | 0.37 | **< 0.001** | -6.85 | 0.40 | **< 0.001** |

A positive β represents a positive correlation between the lung function parameter and resting heart rate
β = The increase in the lung function parameter for every 1 bpm increase in heart rate.
FVC (ml); FEV_1_ (ml)
Basic model (adjusted for age and height)
Adjustment model 1 (age, height, current smoking)
Adjustment model 2 (age, height, current smoking, supine SBP, carotid artery plaques, coronary calcium score, antihypertensive drugs, β-blockers, diabetes, inhalation medication for COPD or asthma)
